# Polyamide 11 Composites with Surface-Activated Intact Mica Structures for Advanced Applications

**DOI:** 10.3390/polym17212861

**Published:** 2025-10-27

**Authors:** Erika Varga, Ferenc Palásti, Attila Bata, Dávid István Kis, Ferenc Tajti

**Affiliations:** Department of Innovative Vehicles and Materials, GAMF Faculty of Engineering and Computer Science, John von Neumann University, 10 Izsáki Street, 6000 Kecskemét, Hungary; palasti.ferenc@nje.hu (F.P.); kis.david@nje.hu (D.I.K.);

**Keywords:** mica, PA11, transcrystallization, SEM, strength

## Abstract

The present study explores the potential improvement of the mechanical properties of bio-based polyamide 11 (PA11) for demanding industrial application using natural and surface-treated mica at 1, 2 and 5 wt%. Suppressed water uptake by up to 4% was revealed with an unfavorable effect of the surface treatment. Impact strength decreased with filler content from 39.6 kJ m^−2^ to between 22–10 kJ m^−2^, while stiffness and resistance towards deformation improved: flexural modulus rose from 518.5 MPa to 596 MPa at 5 wt%-treated small particle, and elastic modulus changed from 542.7 MPa to 705.6 MPa. Particle size dependent trends were observed in crystallinity by Differential Scanning Calorimetry (DSC). Surface treatment promoted the presence of a mesophase form, which was also presented by Scanning Electron Microscopy (SEM). Dynamic Mechanical Analysis (DMA) revealed increased internal friction, temperature-dependent modifications in the elastic properties and a glass transition temperature of 36.6 °C. X-ray Diffraction (XRD) proved an unusual decrease in basal spacing of mica from 9.92 to 9.82 Å due to silanization; however, the compounding process provoked some increase again up to 10.03 Å. Results highlight a viable pathway to modify the properties of PA11 with a primarily role in the filler concentration and dimensions over the surface characteristics.

## 1. Introduction

Polyamides are semicrystalline thermoplastic polymers in which (-CONH-) amid chemical bonds are the fundamental structural elements. All types are widely applied in the industry, with an outstanding role of polyamide 6 (PA6) and polyamide 66 (PA66) [1,2]. Thanks to the high density of the polar functional groups, polyamides possess high strength and durability, good thermal resistance and chemical resistance [3,4], but on the other hand, their UV and O_2_ sensitivity as well as their water uptake, which influences the mechanical properties, can be a disadvantage [5,6]. Polyamide 11 (PA11) is a sustainable, plant oil-based derivative with similar properties to those of polypropylene (PP) and polyamide 12 (PA12), with a more preferable water uptake [7,8,9]. For new solutions at advancing sustainability, high stability at specific circumstances is required from the structural materials [10], thus further improvement of the properties of PA11 is necessary for challenging industrial applications.

The effect of fillers with a high-aspect ratio on polymers has been widely studied [11]. In the paper of Oliver-Ortega et al., PA11 showed high impact strength, which was decreased by natural fibers due to the crack propagation in the interface. On the other hand, well-wetted natural fibers induced lower water diffusion coefficient values thanks to the decreased mobility of the polymer chains, in spite of the increased hydrophilic characteristics [12]. Yu et al. demonstrated that slower cooling of rotomolded PA11 vessel liners results in higher crystallinity degree and consequently improved barrier properties, as well as strength and stiffness [13]. Alo et al. successfully increased flame resistance with the addition of carbon nanofibers and nanoclay to PA11 [14]. Surface-treated and untreated carbon nanofibers increased the elastic modulus and yield strength, and decreased the yield strain and strain at break in a more favorable way after chemical modification [15].

Increased elastic modulus with increasing organo-clay content up to 5 wt% was evidenced by nanoidentification as well [16], accompanied with lower creep values of offshore flexible pipes [17]. Kotek et al. showed that an exfoliated clay structure results in higher stiffness and strength, but toughness is higher at intercalated PA composites [18]. Chow added 4 wt% organically modified montmorillonite (OMMT) to PA6, and observed increased tensile modulus and strength thanks to the extended exfoliation and dispersion of the filler that was proven by X-ray diffraction (XRD) and atomic force microscopy (AFM) [19]. In the study of Kelnar at el., OMMT decreased the size of dispersed EPR (ethene-propene elastomer) and PS (polystyrene)-phase particles in PA6/PS/elastomer nanocomposites, and increased both the strength and stiffness with increasing clay content. Clay particles exfoliated according to the TEM analysis; however, XRD proved intercalation [20].

UV stability of PA11 also was enhanced with the addition of 5 wt% vermiculite [21]. Even non-modified montmorillonite (MMT) exhibited a well-exfoliated structure with significantly increased elastic modulus and thermal stability, when water was injected during the injection phase, thanks to the miscibility of both the clay and the polymer with water [22]. Decorated mica can be utilized in decoration, in sensors and electronic devices as well [23].

Malik tested untreated and treated micas that were covered by silane- or titanate groups in high density polyethylene (HDPE), and detected stronger adhesion between the filler and the matrix above 20% filler addition after surface modification, in which silane compounds were more effective. On the other hand, the application of untreated mica resulted in stress concentration and break [24]. Chen et al. studied the effect of similar particles on the properties on polypropylene (PP). It was revealed that 30% untreated and 40% silanized mica increased the flexural strength by 6.58% and 10.32%, respectively, while at 10% filler concentration the impact strength enhancement was more significant by the untreated version than that silanized one, and the higher values were measured in the presence of titanate surface modification (80.56%). Elongation at break decreased in the presence of mica, which was suppressed after surface treatment [25]. In a recent study partial replacement of carbon black with mica in ethylene–propylene–diene monomer (EPDM)/butadiene rubber (PB) was aimed to reduce the environmental impact. Native and ureidopropyltrimethoxysilane (URE) versions were tested, and it was revealed that with the addition of untreated mica tensile strength, toughness and crosslink density decreased due to the weak connection and gaps, while these properties were enhanced by the surface modified particles [26]. OMMT/PA11 composites showed exfoliation at low concentration, and partly exfoliated partly intercalated structure was confirmed at 6 wt%. OMMT acted as a nucleating agent, and storage modulus increased by 80% as well as tensile strength and strain at break were enhanced, which makes the composite suitable for balloon tubing application [27]. Such increments in storage modulus with glass transition shift were presented by Ismail et al. as well, in PA66 composites, and a significant amount of rigid amorphous phase was also detected in the presence of clay, which enables application as structural components and high temperature parts [28]. In the case of OMMT/aromatic-aliphatic PA composites, the best results in tensile strength and modulus, thermal stability and water uptake properties were obtained at 8 wt% filler concentration, thus utilization in durable components in moisture was advised [29].

Thanks to its balanced mechanical characteristics and sustainability, PA11 has been reported to be a promising candidate for challenging industrial areas such as high-pressure gas containers, including Type 4 hydrogen vessels. In this case, not only the mechanical properties [30,31], but permeation-related phenomena play a key role, as well [32,33]. Failure types of this construction have been summarized recently by Feki et al., Balasooriya et al. and Kis et al., pointing out the needs for improved structural materials [34,35,36]. In present study, we intend to demonstrate a tool to tailor the mechanical properties to adapt PA11 to the actual needs through the addition of mica, a clay studied in compositions with other polyamide derivatives to modify mechanical [37,38], electrical [39], as well as photoresistance and thermal properties [40,41]. In our case, surface-activated mica was produced without any delamination or intercalation, enabling an intrinsic evaluation of the surface behavior. This approach differs from most previous studies where the filler structure was intentionally exfoliated or intercalated; here, the compact multilayer configuration is preserved, allowing the decoupling of surface effects from structural morphology. Hence, the work introduces a novel model system to understand the isolated contribution of surface chemistry on the mechanical and thermal response of PA11. A long-term goal of our research is to provide solution for the pressing problems of hydrogen storage in polymer pressure vessels [42].

## 2. Materials and Methods

### 2.1. Materials

Muscovite mica MU 101 (D_50_ = 17.7 μm, D_95_ = 40.1 μm; referred to as M18) and mica MU 2/1 (D_50_ = 6 μm, D_95_ = 17.4 μm; referred to as M6) were obtained from Imerys S.A. (Paris, France), and Rilsan^®^ Roto 11 PA11 from Arkema S.A. (Colombes, France).

### 2.2. Preparation of the Composites

**Figure 1:**
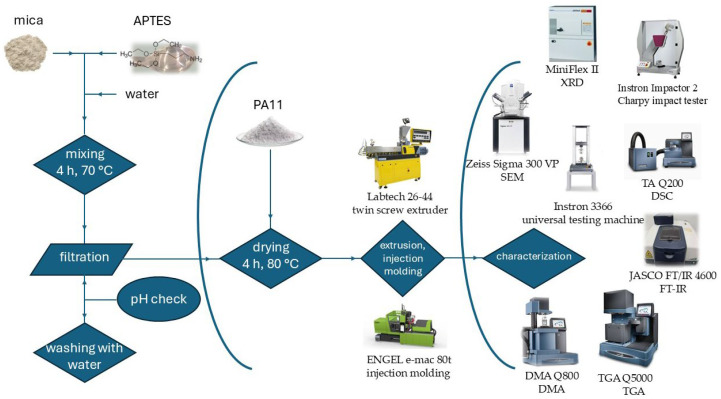
Schematic diagram of material preparation and characterization procedure.

Organo-modified mica samples (designated with T, while untreated mica with UT) were prepared with the addition of 90 wt% DI water and 1 wt% Geniosil APTE (Wacker Chemie AG, Munich, Germany) relative to the weight of mica. The mixture was stirred at 70 °C for 4 h. Mica was then filtrated and washed with distilled water until pH neutrality was obtained (Figure 1).

Raw materials were dried for 4 h at 80 °C before processing. For compounding, a Labtech 26-44 (Labtech Engineering Co., Ltd., Samut Prakan, Thailand) twin screw extruder with a screw diameter of 26 mm and an L/D ratio of 48 was used to prepare composites after drying in dry air at 80 °C for 4 h in a Wittmann Drymax E60 equipment (WITTMANN Group, Kottingbrunn, Austria). Filler contents were 1, 2 or 5 wt%. Screw speed was set to 10 rpm, and zone temperatures varied between 180–210 °C. The air-dried extruded materials were granulated into cylindrical shape. After drying, ISO 527-2 [43] A1 specimens were prepared with an ENGEL e-mac 80t injection molding machine (Engel GmbH., Schwertberg, Austria) for which zone temperatures of 210–180 °C were applied, the mold was tempered at a chilling temperature of 30 °C for 15 s, back pressure was set to 250 bar for 3 min, and a cycle time was 25 s was applied.

### 2.3. Characterization

#### 2.3.1. Density

Immersion method was applied to determine the density of the injection molded samples with a KERN ABT 120-5DM scale (Kern&Sons GmbH, Balingen, Germany) in >99.8% purity methanol obtained from Sigma-Aldrich, St. Louis, MO, USA. Calculated densities were determined based on rule of mixtures using 2.8 g cm^−3^ mica density as indicated on the technical data sheets.

#### 2.3.2. Immersion Water Uptake

Five specimens from each composition with dimensions of 20 × 20 × 2 mm were dried at 80 °C for 48 h, then weighed and immersed in distilled water at 23 or 80 °C. After 2, 24 and 168 h specimens were dried with a wipe and weighed. Water uptake was calculated based on the increase in weight relative to the initial weight. Mean values are presented along with the standard deviations.

#### 2.3.3. Fourier Transform Infrared Spectroscopy (FT-IR Spectroscopy)

FT-IR spectra were recorded with a JASCO FT/IR 4600 instrument (JASCO Corporation, Tokyo, Japan) equipped with an ATR sampling station. An average of 64 measurements was evaluated in the range of 500–4000 cm^−1^ recorded at 4 cm^−1^ steps.

#### 2.3.4. Impact Strength

Notched Charpy impact strength was measured with a Ceast Impactor II instrument (Instron Inc., Norwood, MA, USA) equipped with a pendulum of 15 J according to ISO 179-1 [44] at room temperature. From each composition, at least 5 specimens were tested. Samples were cut from the ISO 527-2 A1 specimens with a length, width and thickness of 80 mm, 10 mm and 4 mm, respectively, and notched with a 6898.000 Intron automatic machine.

#### 2.3.5. Differential Scanning Calorimetry (DSC)

DSC measurements were carried out on 5 mg of granulated samples with a TA Q200 DSC instrument (TA Instruments Inc., New Castle, DE, USA) in high-purity nitrogen gas. Curves were evaluated from the second cycle after removing the thermal history. Heat flow was registered between 30 and 200 °C, and heating rate was set to 10 °C min^−1^ after cooling cycles of 5, 10 and 20 °C min^−1^.

Crystallinity ratio (X_c_) was calculated using Equation (1):

(1) $$X_{c}=\frac{{\Delta H}_{m}}{\left(1-\phi \right){\Delta H}_{m,100}}*100\%$$

where ΔH_m_ is the enthalpy absorbed by the test sample during the melting process, ΔH_m,100_ represents the enthalpy of melting of 100% crystalline polymer and ϕ is the mass fraction of the filler. ΔH_m,100_ of PA11 was taken as 226.4 J g^−1^ [45]. Three specimens were tested from each composition, and the standard deviations were below 0.5%.

#### 2.3.6. Scanning Electron Microscopy (SEM)

SEM images at accelerating voltage of 1 kV were taken on the Charpy-broken specimens with a Zeiss Sigma 300 VP (Zeiss Microscopy, Jena, Germany) instrument equipped with an SE detector. No additional sample preparation was applied.

#### 2.3.7. Dynamic Mechanical Analysis (DMA)

Dynamic Mechanical Analysis (DMA) tests were performed on a DMA Q800 type (TA Instruments Inc., New Castle, DE, USA) measuring device in dual cantilever operational mode, applying a strain amplitude of 15 μm and a frequency of 1 Hz. The scanning temperature ranged from −60 °C to 150 °C and the heating rate was set to 3 °C min^−1^. The test pieces were cut from the ISO 527-2 A1 specimens to a length, width and thickness of 60 mm, 10 mm and 4 mm, respectively. Three specimens were tested from each composition, and the standard deviations were below 0.5%.

#### 2.3.8. Tensile and Flexural Tests

Tensile properties on at least 5-5 ISO 527-2 A1 specimens were measured with an Instron 3366 type universal testing machine (Instron Inc., Norwood, MA, USA) with a crosshead speed of 1.0 mm min^−1^ up to 0.4% extension to determine the elastic modulus, and then at 100.0 mm min^−1^ until the sample was broken at 23 ± 2 °C temperature and 50 ± 10% humidity. To determine flexural properties, 3-point-bending tests were performed using 10 mm min^−1^ bending speed until deflection of 14 mm. Five individual specimens were tested at each composition. Mean values are presented along with the standard deviations.

#### 2.3.9. X-Ray Diffraction (XRD)

To register X-ray diffractograms a MiniFlex II (Rigaku, Tokyo, Japan) instrument using CuK_α_ irradiation (λ = 1.541 Å) was applied at room temperature in the range of 2° to 90°. The X-ray generator was operated at 30 kV with a scan speed of 2° min^−1^. Interplane distance (d_hkl_) and crystallite size were calculated based on the Bragg (2) and Scherrer Equation (3), respectively:

nλ = 2dsin θ(2)

where λ is the wavelength of the incident X-ray, d is the spacing between the parallel planes of atoms in the crystal lattice, θ is the angle of incidence and n is the order of diffraction.

(3) $$\tau =\frac{K\lambda }{\beta cos\;\theta }$$

where τ is the mean size of the ordered crystalline domains, *K* is a shape factor, β is the full width of half maximum of the signal and θ is the angle of incidence.

## 3. Results and Discussion

### 3.1. FT-IR Analysis of Mica

FT-IR analysis can be used to detect whether new functional groups appear on the mica surface after treatment, that would indicate a successful preparation procedure. Mica is a silicate mineral and shows characteristic Si–O bonds in the FT-IR spectrum, while bands corresponding to organic fragments from APTES were expected after silanization.

**Figure 2:**
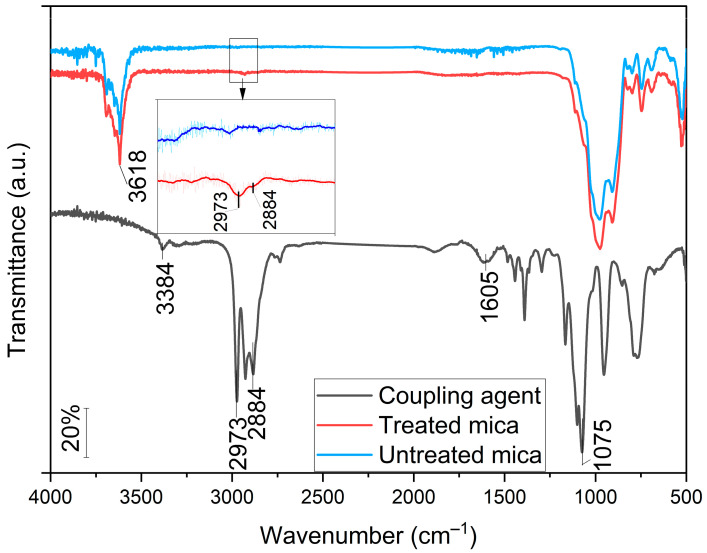
FT-IR spectra of M18, surface-treated M18 and 3-aminopropyltriethoxysilane.

Functional groups on the surface of the treated mica samples were evidenced with FT-IR spectroscopy (Figure 2). Characteristic features of -NH_2_ groups from 3-aminopropyltriethoxysilane were detected at 1605 and 3384 cm^−1^ in the reference sample, which was hardly extinguishable on mica due to decomposition; however, at 2973 cm^−1^ and 2884 cm^−1^, the asymmetric and symmetric stretching vibration of -(CH_2_)- groups could be detected with low intensity, indicating the presence of the organic compounds on the surface, thus it can be concluded that the surface treatment was successful [25,46]. At 1075 cm^−1^ Si-O-C stretching vibration appears [47], while on mica a very intensive transmittance band of -OH at 3618 cm^−1^, and superposition of characteristic vibrations of Si-O Si-O-Al, and Al-O could be observed in the 800–1150 cm^−1^ regime [48].

### 3.2. X-Ray Diffraction

**Figure 3:**
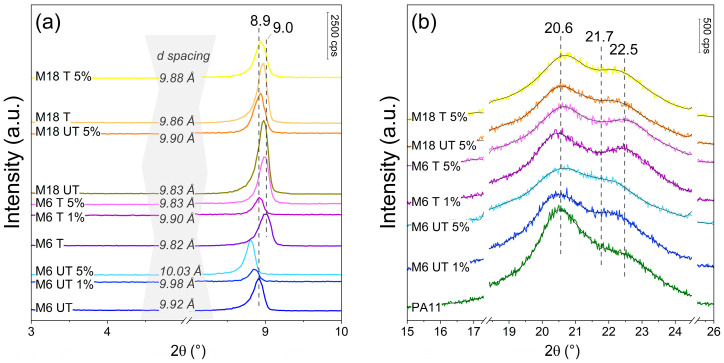
X-ray diffractograms in (**a**) small angle and (**b**) wide angle regions.

In line with former results, before and after silanization, the peak corresponding to the (003) crystal plane of mica was detected at around 8.9° 2θ value, with a little shift of the M18 samples to higher position by 0.04–0.08° (Figure 3a) [49]. Average d spacings between 9.8–10 Å, and crystalline size around 50 nm were determined. As this signal remains intense after silanization, the lack of delamination is proven. Intercalation could not be evidenced either, as no significant shift to lower 2θ appeared, which could be evenly manifested in a peak position of 4–5° [50], and calculated layer spacing values differed by a maximum of 0.1 Å [51]. Unusually, after surface treatment, the distance of the crystal sheets decreased in the case of M6 composites, which can be the result of the removal of some cations by water from the interplanar space during the reaction and multiple washes, followed by crystal relaxation during drying [52]. These findings support that mica structure in these circumstances is stable and resistant towards expansion. However, in all cases, crystal plane space increased during compounding regardless of the surface treatment or particle size. In addition, in composites with natural mica, the changes are more pronounced, probably due to the less compact initial structure. Increasing plane distance is a sign of a weak interaction between the matrix and the filler during dynamic processes. Smaller particles are more sensitive to this impact, which is indicated by larger changes, and it is less significant at organo-modified surfaces. This phenomenon might be attributed also to the role of the polar groups in PA11 in the incorporation of inorganic particles with high oxygen moiety on the surface.

Small-intensity diffraction peaks at 20.6 and 22.5° in Figure 3b correspond to the (100) and (110)/(010) reflections of the α’ crystalline form of PA11, respectively, with a significant contribution of a component at about 21.7 2θ value that indicates the presence of pseudohexagonal γ form even in the case of the neat PA11 [53,54]. The addition of mica resulted in a decrease of the former peak, which was more significant at higher concentrations. This is in line with the DSC results and the observations of Yoon et al., who showed that α-phase of PA6 can only form at a distance from the silicate surface where the ability of the chains to fold is not interrupted [55].

Although DSC measurements suggested an increased γ/α ratio in certain samples, this tendency cannot be clearly resolved from XRD, because the γ-phase reflections of PA11 overlap with those closely spaced reflections of the α-phase around 21–22° 2θ, and their low intensity prevents quantitative identification. In contrast, enthalpic changes from polymorphic transitions captured by DSC are more sensitive to the minor γ or mesophase formation. Therefore, the DSC results indicate thermodynamic evidence for γ-phase presence that XRD cannot clearly confirm under these experimental conditions.

### 3.3. Differential Scanning Calorimetry

**Figure 4:**
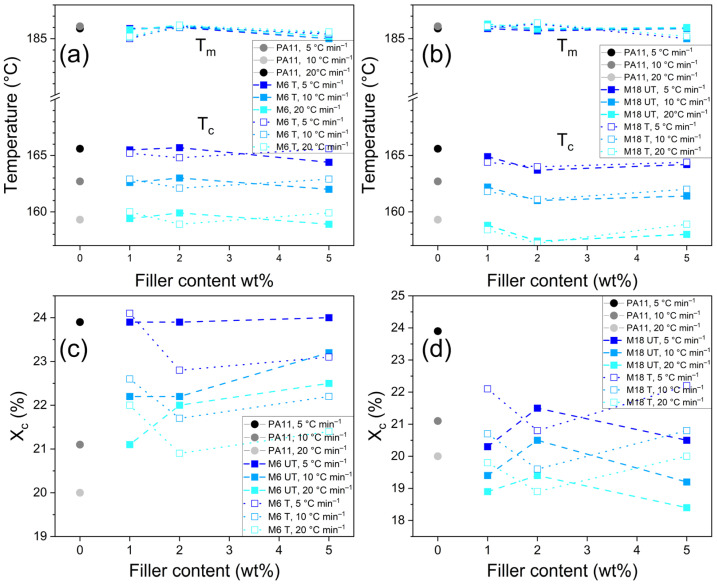
T_c_ and T_m_ results of (**a**) M6 and (**b**) M18 composites, as well as X_c_ values of (**c**) M6 and (**d**) M18 composites after different cooling cycles.

**Figure 5:**
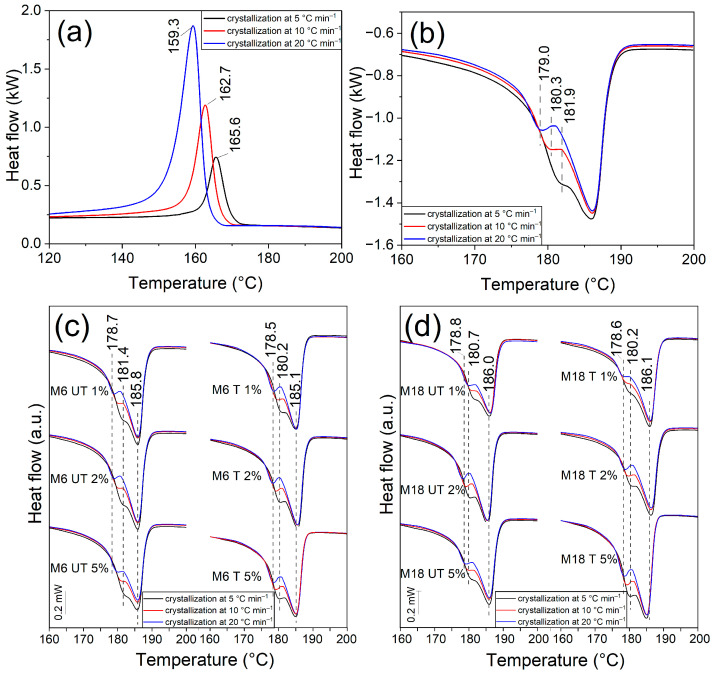
DSC curves of (**a**) PA11 at crystallization and (**b**) PA11 melting, and melting DSC curves of composites with (**c**) M6 and (**d**) M18 particles.

Differential Scanning Calorimetry (DSC) is suitable to determine the crystallinity ratio, the melting point, the glass transition temperature and the crystallization temperature, by which the thermal stability and the processability can be described, as well as mechanical properties can be anticipated. DSC curves showed crystallization peaks with increasing intensity and decreasing crystallization temperature (T_c_) at increasing cooling rate, as well as a double melting peak in all cases, including the neat polymer (Figure 4 and Figure 5a,b). The former phenomenon can be attributed to the shorter time for crystallization at higher temperatures and faster crystalline formation. Peak maximum of T_c_ was located at somewhat lower temperatures at the composites, especially in the case of M18 addition, regardless of the surface treatment. This means that the material solidifies faster, which shortens the cycle period of the processing. In the case of melting temperature (T_m_) peak maxima, such correlations could not be observed.

With increasing the cooling rate, the total crystallization degree decreased. After analyzing the results thoroughly, a dependence on the filler particle size could be evidenced: for M18 samples, the total crystallization ratio was lower compared to that of the neat PA11, which is in line with our previous result on polyethylene (PE) composites of 10 wt% natural mica with 15 μm D_50_ value [56]. The effect of the surface treatment is inconsistent among the samples with different filler loadings. In the presence of untreated M6 particles, however, an increment in X_c_ can be detected, which was more significant at higher cooling rates. Treated M6 particles at 2 wt% and 5 wt% concentration induced a smaller crystalline ratio than the untreated version, but still higher than M18. At 5 °C min^−1^, the values were smaller than those of the neat PA11.

DSC curves in Figure 4c,d reveal that the formation of a crystalline form with lower melting temperature is different in case of the surface-treated and untreated fillers. The intensity of this minor component decreases with increasing cooling rate, as well as the peak maximum shifts by approximately 2 °C to lower temperatures, which was more pronounced in case of untreated M6 particles. It has been shown previously that quenching from the melt enhances γ crystallization, and mica increases α-phase crystallization rate of PA6 and PA6,6 composites [57], while Venkataramani reported that increasing clay content increases transformation of α to γ form of PA6/PA66 composites, and increases the crystallization temperature thanks to a strong heterophase nucleation effect [58]. In the research of Uno et al. on mica/PA composites, only the mixtures had lower melting points, with a weak shoulder around 210 °C that was attributed to the formation of γ crystalline form [59]. In another study, PA11 and its diatom composites showed two melting peaks, which rather appeared as intense shoulders at high diatom content as of 5%, and it was suppressed at 20 wt%. This feature at around 182 °C was attributed to the formation of γ crystalline phase (smectic phase), accompanying the α-phase melting at about 189 °C [60]. In our case, side peak can indicate the presence of either γ form or mesophase.

It has also been reported that in situ polymerization of PA11 in the presence of clay results in increased crystallization rate due to the supernucleating effect of the nanolayers, while after isothermal crystallization, two melting peaks appeared: the one at the lower temperatures was attributed to the melting of the crystals formed during isothermal crystallization, and its intensity increased with increasing T_c_, while the peak at the higher temperatures was constant and originated from recrystallization. Small crystallites were detected in the nanocomposites with Small Angle Light Scattering (SALS) [61]. A bimodal structure at non-isothermal characterization due to recrystallization was presented by Verkinderen et al. as well [62]. Similar nucleating effects and finer structure of crystals were detected by Jariyavidyanont et al. in the case of sepiolite and organo-modified MMT (OMMT) composites of PA11 at fast cooling; they also concluded that not only the number the nucleation sites but also the segment mobility constraints play an important role [63].

The results in Figure 5 indicate that with increasing the cooling rate, the ratio of the two crystalline forms changes in favor of the α-phase, and the peaks corresponding to melting of the γ phase can be distinguished better. In another study, at fast cooling, mesophase was formed instead of part of the crystals in PA11, while in the presence of OMMT, the crystallization temperature increased above 10 K s^−1^ cooling rate, but crystallization was not suppressed. At low crystallization rate, however, the peak was practically unchanged, which was different in the case of neat PA11. Mesophase was not detected in case of the composites, which is in contrary to our observations [64].

The present study revealed that in both the cases of M6 and M18 the increment of the filler content or surface treatment resulted in an enhanced γ-to-α-phase ratio (Figure 5c,d). Chain movement in the γ form is less restricted, thus the polymer phase becomes less fragile according to the DSC analysis. In former research, a slight increase in the crystallization temperature could be observed without correlation with silanization; however, difference in selectivity towards α or γ polymorph was proven by X-ray Diffraction (XRD) [60].

### 3.4. Densities and Water Uptake

**Figure 6:**
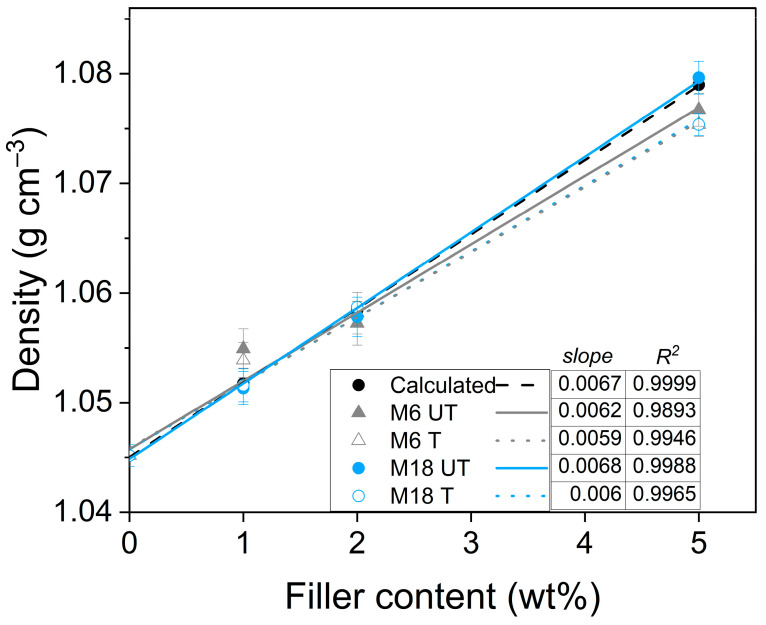
Densities of neat PA11 and composites with trend lines.

Measured densities are depicted in Figure 6. In line with the expectations, by increasing the filler content, densities increase following a linear trend. Based on the small deviations from the calculated data, it can be concluded that the composites have compact structures; however, considering the consistently lower values, the structure in case of the surface modified mica is less compact. On the other hand, larger untreated particles prevent void formation [65].

**Figure 7:**
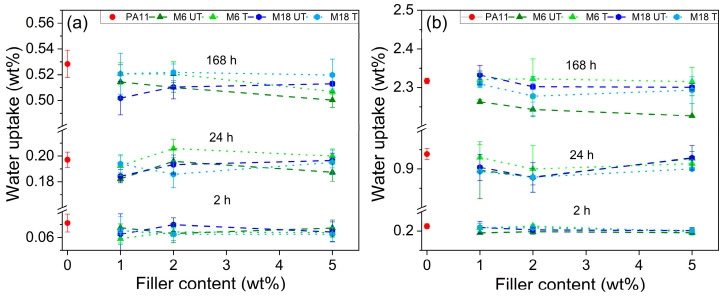
Water uptake at (**a**) 23 °C and at (**b**) 80 °C.

Water uptake results after soaking time of 2, 24 and 168 h are represented in Figure 7. For neat PA11 after 2 h at 23 °C, 0.06–0.07 wt% of water was absorbed, which increased to around 0.55% after 168 h at 23 °C, while it was enhanced to 0.2 and 2.3 wt% at 80 °C. The addition of mica decreased these values. After 168 h some trends can be observed: M6 particles induced a decreasing water uptake with increasing filler content at both temperatures. M18 composites showed similar but suppressed trends at elevated temperature but an increment at room temperature. At both particle sizes, however, surface treatment seems to be somewhat unfavorable. Based on these results, it can be concluded that the larger impermeable particle measures increase the filler–matrix interface surface, which is imperfect regarding permeation, thus prone to pass water.

Fluid absorption of polyamides and their composites is a key issue in the everyday engineering [66,67]. Vlasveld et al. showed that clays slow down the water absorption rate of PA6; however, equilibrium water content is similar, indicating that water is absorbed by not only the amorphous phase of PA6, but in the surfactant on the filler as well, which is in agreement with our findings [68].

### 3.5. Scanning Electron Microscopic Analysis

**Figure 8:**
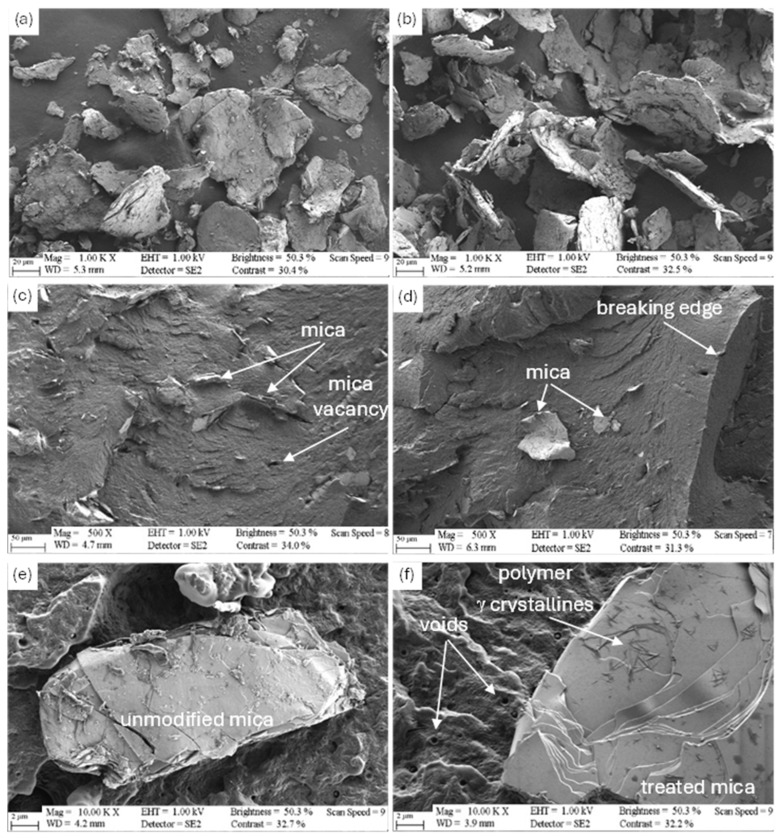
SEM images of (**a**) untreated and (**b**) treated M18, (**c**) M18 UT 5% and T 5% (**d**) samples and (**e**) M6 UT 1% and (**f**) T 1% samples.

SEM images in Figure 8 show mica platelets embedded in the PA11 matrix. Interfacial debonding and voids (processing-induced air bubbles or debonding gaps) are visible. Breaking edges and sharp fracture zones indicate predominantly brittle fracture behavior. Preliminary research on the lamellar mica particles did not show any structural modification on the mica samples due to the surface treatment, as depicted in Figure 8a,b, and delamination to platelets with nanometer thickness during compounding neither could be evidenced (Figure 8c,d). Agglomeration-free distribution and orientation parallel to the injection flow can be seen in Figure 8c,d regardless of surface modification. Taha et al. presented that agglomerated MMT in different matrixes reduces the mechanical properties; however, after re-processing, better dispersion and reinforcement can be achieved [69]. Based on these observations, homogeneous distribution of inorganic particles in the polymer matrix is mainly driven by the processing conditions rather than the surface chemistry.

Studying the inorganic particles at high magnification within the composites, smooth unmodified surfaces were observed in case of natural mica, in contrast, features indicating a crystallization process on the modified surface can be seen in Figure 8f. Although the precise structural origin of these features cannot be clearly confirmed, similar behavior has been widely reported for polymer–clay systems. Dasari et al. showed by Transmission Electron Microscopy (TEM) that silicate monolayers of the modified clay can act as nucleation sites for 30–40 nm thick transcrystalline layer, in which the bonding of the ammonium cations of PA6 to the surface might play an important role [70]. Anoukou et al. demonstrated that the crystalline phase composition of polyamide 6 is altered in the area of silicate surfaces, forming a so-called interphase region enriched in γ-form crystals [71]. This observation supports the concept that the clay surface can promote a distinct crystallization mode. While the evidence is consistent with surface-induced crystallization, the interpretation suggests that overlapping phenomena, such as subtle interfacial ordering or local variations in crystallinity, may also contribute. In the study conducted by Chen et al., well-organized transcrystallization with parallel structure was detected on silanized mica surfaces in etched HDPE composites, which phenomenon was significant only at lower matrix density [72]. In the present case, however, surface structures are intercrossing and have lower density, based on which a less active surface and different mechanism is proposed. On the other hand, this form probably contributes to the minor peak on the DSC melting curves.

### 3.6. Mechanical Testing

**Figure 9:**
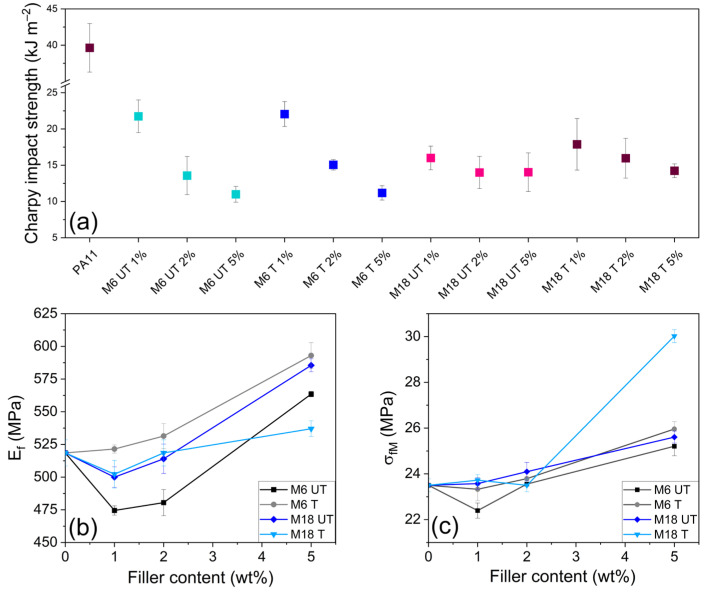
(**a**) Charpy impact strength, (**b**) concentration-dependent flexural modulus and (**c**) maximum flexural stress results on PA11 and composites.

Notched Charpy impact tests on the composites at room temperature revealed a significant decrease in absorbed energy before breaking in all cases, which was increasing with increasing the filler loading. In addition, a dependence on the filler size and surface treatment can also be detected in Figure 9a. Original PA11 impact strength of 39.64 kJ m^−2^ decreased to 21.73, 13.57 and 10.99 kJ m^−2^ after the addition of 1, 2 and 5 wt% of untreated M6, respectively. Treated M6 particles provoked slightly smaller decrease at all loadings. This trend was similar in the case of the larger particles with lower values at 1 wt% loading but higher ones at 2 and 5 wt%.

Flexural test results are presented in Figure 9b,c. M6 T particles increased the flexural modulus with increasing the filler concentration, while in case of the untreated and larger versions a drop could be observed first, that changed to improvement at 5 wt% compared to the neat PA11. This is probably provoked by microvoid formation that facilitates heat dissipation deformation mechanisms, transfer of stresses between polymer and nanofiller and by splitting, opening and slippage of clay layers [38]. A positive effect of the silanization was significant at M6 filler, while at M18 difference was detected only at loading of 5 wt% in a declining manner. Results on M18 were higher than on M6 untreated particles, but these were lower than in case of M6 after modification. Regarding maximum bending stress (Figure 9c), an increment can be observed with increasing filler content after a stagnation or decrease at 1 wt%, clear principles of which are hardly distinguishable.

Fornes and colleagues observed greater clay exfoliation in PA6 than in PA11 and PA12. They also found that PA6 exhibited Izod impact strength that remained consistent regardless of concentration, whereas in PA11 and PA12, impact strength decreased with increasing filler content. This behavior was attributed to differences in the ductile-to-brittle transition temperatures [73]. In another study on mica and glass fiber-reinforced PA6 composites, impact strength declined with increasing mica content, while tensile and flexural strengths remained largely unaffected [74]. In both PA12 and PA6-PA66, copolymer notched impact strength decreased by modified clay; however, crystallization was enhanced in PA6/6,6, but unchanged in PA12, as well as large increment occurred in strength, modulus and heat deflection temperature (HDT), which was attributed to the oriented exfoliated clay layers with compact lamellar crystals on them [75]. Based on our DSC and SEM examinations, the size-dependent defect-forming property of mica is counterbalanced by an enhanced filler–matrix connection appearing by recrystallization. This effect correlates with the available surface of mica, thus showing dependence on the concentration and the particle size.

**Figure 10:**
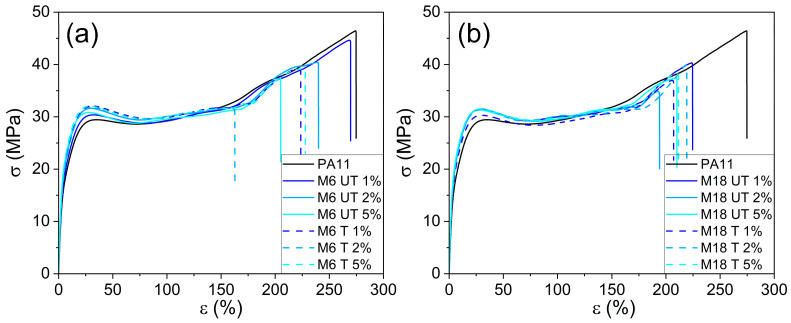
Representative tensile curves in case of M6 (**a**) and M18 (**b**) composites.

**Figure 11:**
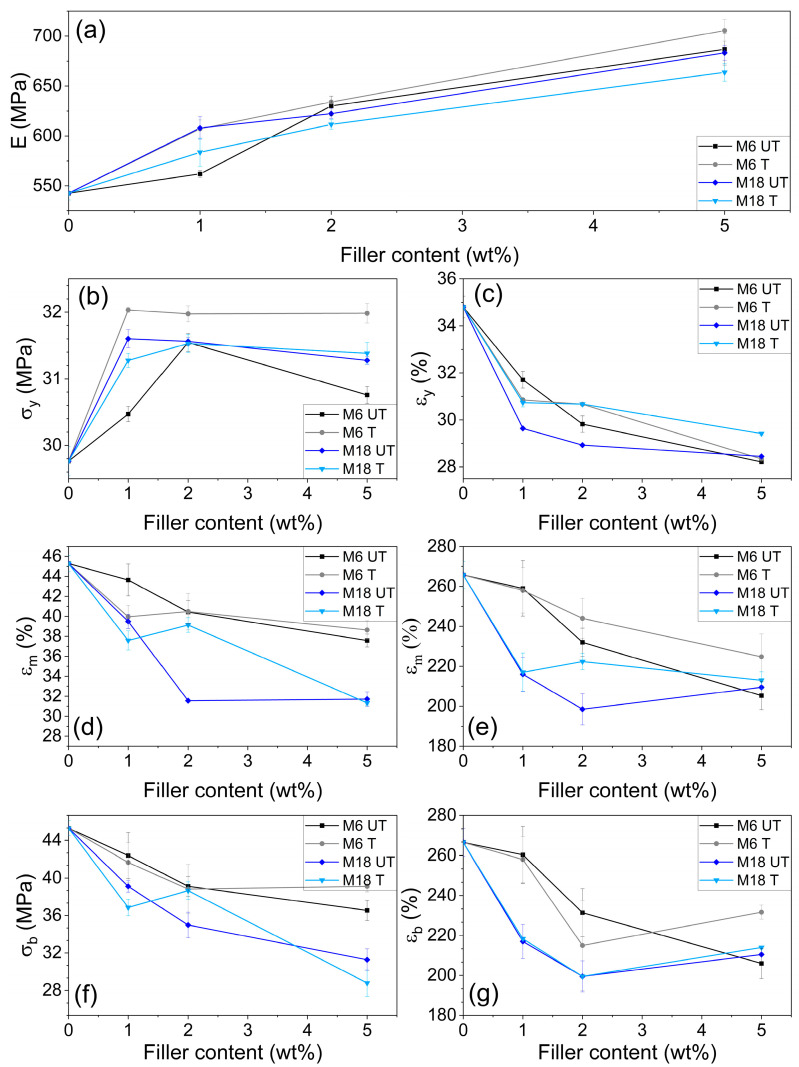
Filler concentration dependence of (**a**) elastic modulus, (**b**) stress at yield and (**c**) strain at yield, (**d**) stress at maximum load and (**e**) strain at maximum load, (**f**) stress at break and (**g**) strain at break.

One measured tensile stress–strain curve is depicted in Figure 10 for each sample type. A well-detectable yield point was observed in all cases; however, the characteristics of the curves were the same. Results at high elongation might not have relevance in application and can show high deviation, thus mean values are presented in Figure 11 with the indication of the standard deviances of parallel measurement results.

Elastic moduli in Figure 11a demonstrate an increasing trend with the filler concentration in all cases starting from the result of neat PA11 of 542.7 MPa. With smaller filler particles higher values were achieved with a maximum of 705.6 MPa. Comparing the two different particle sizes, surface treatment provoked opposite trends, which can be attributed to the multiple effects of the rigid filler volume in the material, and the quantity of the chemically provoked filler–matrix joining points. Similarly to that, Masenelli-Varlot et al. reported that elastic modulus depends mainly on the clay loading after studying intercalated and exfoliated composites of MMT PA6, and this effect was dependent on the filler orientation. On the other hand, in low-deformation tests no significant difference between the two types of fillers was evidenced [76]. In contrast, Mahanwar et al. presented that the elastic modulus was higher than the predicted values when tetra-isopropyl titanate was used as adhesion promoter in mica/PA6 composites [77]. Homogenization theories revealed that characteristic results on elastic moduli and thermal expansion coefficient appears for flake or oblate fillers at random orientation as well [78].

Regarding stress at yield results (Figure 11), it can be concluded that silanization induced significant increment in the values only at M6. After a rise at 1 wt% content, further filler addition induced a minor decrease in all the cases. This critical concentration was above 2 wt% at M6 UT, and in smaller scale at M18 T samples. Strain at yield decreased in the presence of fillers: after adding more than 1 wt% mica, the slope of the curves in Figure 11c was less steep in case of M18, and in this case silanization resulted in larger difference in favor of the composites with the treated version.

In Figure 11d,e, stress and strain at maximum load results are depicted. In the former case, the value of 45.3 MPa at PA11 decreased with increasing the filler loading accompanied by a clear dependence on the filler size rather than on the surface chemistry; in the case of M18 the values are approximately 30% lower at 5 wt% mica concentration compared to the original value, while at M6 the difference does not exceed 18% regardless of silanization.

Stress at break (Figure 11f) decreased in the presence of increasing amount of mica. The effect of the surface treatment is not consistent; however, the particle size has a more pronounced influence on the results: at 5 wt% filler content σ_b_ was 36.5 MPa for M6 UT and 31.3 MPa for M18 UT samples, respectively. Strain at break (Figure 11g) showed similar behavior.

In contrast to our observations, when mechanical properties of PA6/glass fiber and mica composites were compared by Ünal et al., the fillers increased the rigidity; a linear increment in tensile strength and tensile modulus was detected in case of glass fiber addition, but these properties were nearly independent of the mica addition [79]. It was proven by Liu et al. that higher clay concentration than 4 wt% results in less extended exfoliation and dispersion and enhances less the mechanical properties (evidenced by XRD, DMA, TEM, tensile tests), and decreases the thermal stability [80]. Similar concentration-dependent exfoliation was detected by Risite et al., as well as improvement in tensile and thermal properties [81]. Bureau et al. concluded that the reinforcing effect of MMT in PA6 regarding rigidity and strength was the result of the reinforcing effect of the filler rather than the crystalline structure [82]. Kis et al. similarly demonstrated a linear increase of elastic modulus with increasing filler contents in OMMT/PA6 systems, and highlighted the improved yield strength, as well as an optimal filler loading of 1 wt% regarding stiffness-brittleness balance, which is very similar to our observations [83]. Partly differing from these results, in biodegradable composites, OMMT provoked a clear concentration-dependent increase in the elastic modulus, and decrease in the crystallization rate, yield strength and impact strength [84].

Present test results support the observations on ideal microstructure model of Masenelli-Varlot, where a key role in enhancing the properties was attributed to the avoidance of agglomeration and the three-dimensional network rather than to the complete exfoliation of the filler [85].

### 3.7. Thermomechanical Testing

**Figure 12:**
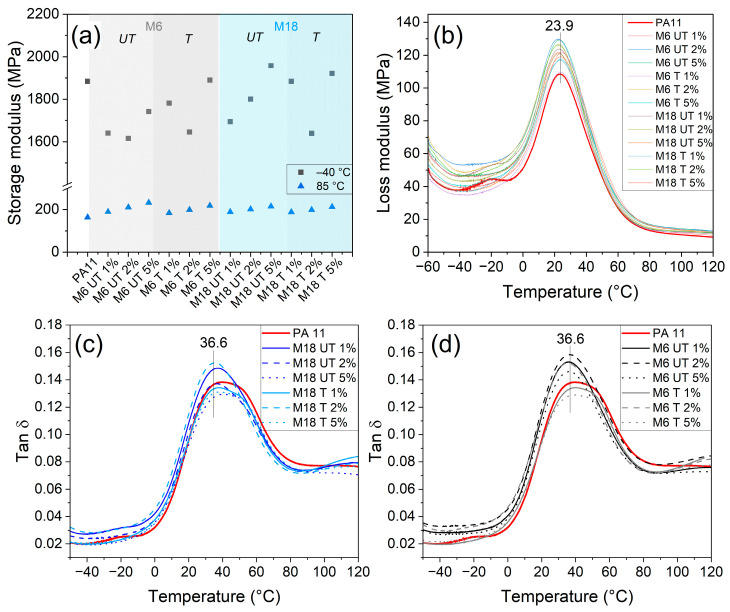
DMA analysis: (**a**) specific storage moduli values, (**b**) loss moduli, (**c**) tan δ results of M6 and (**d**) tan δ results of M18 composites.

As proven by DMA, storage moduli decreased continuously in all cases with temperature. Values at −40 and 85 °C are presented in Figure 12a. At −40 °C, results randomly decreased compared to PA11; however, at 85 °C a correlation with the filler concentration can be observed. This indicates that the quantity of the rigid particles influences the viscoelastic properties above T_g_ in line with the tensile test results of the elastic modulus. Phenomena similar to the results of Vlasveld et al., who presented that the storage modulus of PA6 composites increased at higher degree after addition of modified synthetic mica due to the enhanced exfoliation and more individual particles, could not be detected [86].

Location of the loss modulus peak maximum did not change from 23.9 °C after compounding, but the intensity increased with the addition of fillers in a random way. This feature can be identified as α relaxation corresponding to the movements of the main segments in the amorphous phase. In the study of de Salles Macena da Cruz et al., graphene nanoparticles reduced the loss modulus, which is in contrary to present observations [87]. However, enhancement in both storage and loss moduli meets our previous results on mica/PE composites without the prominent role of mica with higher aspect ratio, resulting from the presence of rigid fillers and restricted chain motions [56]. Increment in loss modulus and thus tan δ at T_g_ is the indication of homogeneous distribution of the reinforcing agent resulting in higher number of mobile chain segments and internal friction.

Glass transition temperature of around 36.6 °C was determined based on the peak maximum of tan δ. Instead of a sharp feature, even in the case of the neat PA11, a broad, apparently multicomponent peak was detected. Comparing that with DSC curves, this indicates the coexistence of amorphous phases in the neighborhood of both the α and γ crystalline forms. The rise in T_g_ could be an evidence of polymer clay interactions [38]; however, after the addition of fillers, the component at the higher temperature disappeared mainly in the cases of the untreated fillers. Smaller particles were more effective in suppressing the component at lower temperature.

As observed by Dobrosielska et al., in the case of diatomic/PA11 composites, silanization resulted in limited T_g_ modification, which indicated less influence on chain movement, as well as on elastic modulus and tensile strength. Higher storage modulus showed increased stiffness and improved viscoelastic performance. At 20 wt% filler content, a decrease in tan δ values was observed through reduced polymer relaxation due to hindered mobility [60]. These explanations meet well with the present results. Based on DMA results, it can be concluded that surface chemistry plays a minor role in comparison with the concentration of the rigid fillers, and the formation of a mesophase broadens the glass transition temperature regime, which depends on the actual composition.

## 4. Conclusions

The present study investigated how mechanical, thermal and thermomechanical properties of PA11 are influenced by inorganic lamellar fillers. Unlike previous works on exfoliated or nanostructured clays, this study introduces a novel concept of using intact, non-delaminated mica with surface activation to reveal the independent role of interfacial chemistry. This experimental design enables a clearer understanding of how particle dimensions and concentration govern property enhancement in bio-based PA11 composites.

Organically modified mica samples were prepared with retaining the compact multilayer structure that was revealed by the basal spacings of around 9.9 Å. On the other hand, FT-IR spectra indicated the presence of -CH_2_- groups originating from the organic modifier at 2973 and 2884 cm^−1^. Our results highlight that filler concentration plays a key role in the overall performance of the composites. In case of the impact strength, the original 39.6 kJ m^−2^ decreased by 45% and 73% at 1 and 5 wt% M6 T content, respectively, while for M18, the concentration dependence was less significant. Prolonged water uptake was suppressed by mica at room temperature, and at 80 °C 1 wt% M6 UT particles were the most effective. The addition of mica resulted in increased shape retention; E_f_, however, decreased at lower concentration, then at 5 wt% increased by up to 27% compared to that of the neat polymer. Elastic modulus increased continuously with the filler concentration from 543 to 660–690 MPa. Stress at yield was almost constant at increased values between 31–32 MPa in case of the composites, while strain at yield was lower by an average of 15%. These changes are attributed to the presence of the rigid fillers in the polymer matrix. Strain and stress at maximum load as well at break decreased with increasing filler content. DMA demonstrated that energy dissipation and thermomechanical stability improved after filler incorporation in a temperature-dependent manner, without correlation with the surface treatment. Tg of 36.6 °C was the same in case of all the samples.

DSC revealed slightly decreased total crystallinity in case of larger particles by a maximum of 3.6%, and in contrast, mica with smaller D_50_ enhanced crystallization by up to 2.5%. An increment in a mesophase/γ form content was detected in case of surface-treated mica, while SEM indicated polymer crystallization on treated mica surfaces. Despite these observations, changes in total crystallinity did not directly correlate with the mechanical performance, suggesting that the filler–polymer interfacial interactions and physical barrier effects play a critical role.

XRD analysis showed an unusual reduction in crystal plane spacing after surface modification of mica from 9.92 to 9.82 Å, which was then compensated during melt compounding. However, in neither case could delamination be detected. These findings confirm that compounding of PA11 with mica enable tailoring properties for targeted industrial applications, including structural components and pressure vessel liners in hydrogen storage systems. This work contributes to the broader field of sustainable high-performance polymers by offering new insights into structure–property relationships and processing performance trade-offs in PA11-based composites.

The enhanced stiffness, thermal stability and dimensional integrity of the composites make the studied composites promising candidates for lightweight structural components in the automotive and aerospace sectors. Moreover, the combination of thermal resistance, barrier effect and mechanical strength suggests potential for hydrogen storage system components, such as pressure vessel liners. These materials can be utilized in pipeline coatings, electrical housings and eco-designed consumer goods as well, where reduced environmental footprint and long service life are required. Optimizing the interfacial chemistry between PA11 and inorganic fillers to achieve a balanced combination of stiffness, toughness, and durability are interesting topics for future research. Validation of the composites through long-term studies on aging, hydrogen permeability and large-scale processing for sustainable industrial applications are also desirable.

## Data Availability

The original contributions are included in this article. Further inquiries can be requested from the corresponding author.

## Abbreviations

The following abbreviations are used in this manuscript:

DSC	Differential Scanning Calorimetry
DMA	Dynamic Mechanical Analysis
FT-IR	Fourier Transform Infrared Spectroscopy
SEM	Scanning Electron Microscopy
MMT	Montmorillonite
XRD	X-ray Diffraction
PA	Polyamide

## References

[1] Pervaiz M., Faruq M., Jawaid M., Sain M. (2017). Polyamides: Developments and Applications Towards Next-Generation Engineered Plastics. Curr. Org. Synth..

[2] Kondo M.Y., Montagna L., Morgado G., Castilho A., Batista L., Botelho E., Costa M., Passador F., Rezende M.C., Ribeiro M. (2022). Recent Advances in the Use of Polyamide-Based Materials for the Automotive Industry. Polímeros.

[3] Sonawane S.S., Mishra S., Shimpi N.G. (2009). Polyamide Nanocomposites: Investigation of Mechanical, Thermal and Morphological Characteristics. Polym.-Plast. Technol. Eng..

[4] Cruz P., Shoemake E.D., Adam P., Leachman J. (2015). Tensile Strengths of Polyamide Based 3D Printed Polymers in Liquid Nitrogen. IOP Conf. Ser. Mater. Sci. Eng..

[5] Gijsman P., Meijers G., Vitarelli G. (1999). Comparison of the UV-Degradation Chemistry of Polypropylene, Polyethylene, Polyamide 6 and Polybutylene Terephthalate. Polym. Degrad. Stab..

[6] Song J., Ehrenstein G.W. (1990). The Influence of Water Absorption on the Properties of Polyamide. Kunstst. Ger. Plast..

[7] Jariyavidyanont K., Focke W., Androsch R. (2019). Thermal Properties of Biobased Polyamide 11. Thermal Properties of Bio-Based Polymers.

[8] Bahrami M., Abenojar J., Martínez M.A. (2021). Comparative Characterization of Hot-Pressed Polyamide 11 and 12: Mechanical, Thermal and Durability Properties. Polymers.

[9] Oliver-Ortega H., Granda L.A., Espinach F.X., Delgado-Aguilar M., Duran J., Mutjé P. (2016). Stiffness of Bio-Based Polyamide 11 Reinforced with Softwood Stone Ground-Wood Fibres as an Alternative to Polypropylene-Glass Fibre Composites. Eur. Polym. J..

[10] Surisetty J., Shahroodi Z., Lucyshyn T., Oreski G. (2025). The Effect of Liquid Organic Hydrogen Carrier on the Physico-Mechanical and Rheological Properties of Injection-Molded High-Density Polyethylene and Polyketone. Results Eng..

[11] Gyawali B., Haghnazar R., Akula P., Alba K., Nasir V. (2024). A Review on 3D Printing with Clay and Sawdust/Natural Fibers: Printability, Rheology, Properties, and Applications. Results Eng..

[12] Oliver-Ortega H., Méndez J.A., Espinach F.X., Tarrés Q., Ardanuy M., Mutjé P. (2018). Impact Strength and Water Uptake Behaviors of Fully Bio-Based PA11-SGW Composites. Polymers.

[13] Yu M., Qi L., Cheng L., Min W., Mei Z., Gao R., Sun Z. (2023). The Effect of Cooling Rates on Thermal, Crystallization, Mechanical and Barrier Properties of Rotational Molding Polyamide 11 as the Liner Material for High-Capacity High-Pressure Vessels. Molecules.

[14] Lao S., Wu C., Moon T., Koo J., Morgan A., Pilato L., Wissler G. (2009). Flame-Retardant Polyamide 11 and 12 Nanocomposites: Thermal and Flammability Properties. J. Compos. Mater..

[15] Mancic L., Osman R.F.M., Costa A.M.L.M., d’Almeida J.R.M., Marinkovic B.A., Rizzo F.C. (2015). Thermal and Mechanical Properties of Polyamide 11 Based Composites Reinforced with Surface Modified Titanate Nanotubes. Mater. Des..

[16] Hu Y., Shen L., Yang H., Wang M., Liu T., Liang T., Zhang J. (2006). Nanoindentation Studies on Nylon 11/Clay Nanocomposites. Polym. Test..

[17] Rodrigues A., Bastos I., Abud Kappel M.A., Nascimento C., Ferreira L., Silva A. (2021). Micromechanical Property Study of Nylon 11 and Organoclay Systems for Offshore Flexible Pipe. Fibers Polym..

[18] Kotek J., Baldrian J., Slouf M. (2008). Deformation and Fracture Behavior of Polyamide Nanocomposites: The Effect of Clay Dispersion. J. Appl. Polym. Sci..

[19] Chow W.S. (2007). Mechanical, Morphological and Rheological Properties of Polyamide 6/Organo-Montmorillonite Nanocomposites. Express Polym. Lett..

[20] Kelnar I., Rotrekl J., Kotek J., Kaprálková L., Hromádková J. (2009). Effect of Montmorillonite on Structure and Properties of Nanocomposite with PA6/PS/Elastomer Matrix. Eur. Polym. J..

[21] Kaci M., Dehouche N., Focke W., van der Merwe E. (2019). A Degradation Study of Polyamide 11/Vermiculite Nanocomposites under Accelerated UV Test. Polym. Eng. Sci..

[22] Stoclet G., Sclavons M., Devaux J. (2013). Relations between Structure and Property of Polyamide 11 Nanocomposites Based on Raw Clays Elaborated by Water-assisted Extrusion. J. Appl. Polym. Sci..

[23] Zhu Q., Chua M.H., Ong P.J., Cheng Lee J.J., Le Osmund Chin K., Wang S., Kai D., Ji R., Kong J., Dong Z. (2022). Recent Advances in Nanotechnology-Based Functional Coatings for the Built Environment. Mater. Today Adv..

[24] Malik T.M. (1991). Morphological and Mechanical Studies of Surface Treated Mica Reinforced High Density Polyethylene. Polym. Bull..

[25] Chen X., Zhang T., Sun P., Yu F., Li B., Dun L. (2022). Study on the Performance and Mechanism of Modified Mica for Improving Polypropylene Composites. Int. J. Low-Carbon Technol..

[26] Jung W.-Y., Cho S.-W., Jang K.-S. (2025). Effect of Surface-Modified Mica in Hybrid Filler Systems on the Curing and Mechanical Behavior of Ethylene–Propylene–Diene Monomer (EPDM)/Butadiene Rubber (BR) Blend. Polymers.

[27] Halim K.A.A., Farrell J.B., Kennedy J.E. (2013). Preparation and Characterisation of Polyamide 11/Montmorillonite (MMT) Nanocomposites for Use in Angioplasty Balloon Applications. Mater. Chem. Phys..

[28] Ismail M.A., Yousef E.A., Nasr G.M. (2025). Surface Modification of Montmorillonite—MMT Nanofiller: How It Affects Both the Rigid Amorphous Fraction (RAF) and the Physical Properties of Polyamide 66. Polym. Bull..

[29] Zulfiqar S., Ahmad Z., Ishaq M., Sarwar M.I. (2009). Aromatic–Aliphatic Polyamide/Montmorillonite Clay Nanocomposite Materials: Synthesis, Nanostructure and Properties. Mater. Sci. Eng. A.

[30] Li X., Dong C., Liu Y., Li J., Bin G., Zhou C., Han W. (2024). Study on the Effect of Hydrogen Cycle Pressure Relief Time on the Hydrogen Permeability and Mechanical Properties of Polyamide Liner Materials for Type IV Hydrogen Storage Cylinders of HFCVs. Int. J. Hydrogen Energy.

[31] Cheng L., Qi L., Tang X., Li X., Chen L., Min W., Mei Z., Gao R., Sun M., Xiao J. (2024). Effects of Hydrogen Cycling on the Performance of 70 MPa High-Pressure Hydrogen Storage Tank Liners Formed by Different Processes. Int. J. Hydrogen Energy.

[32] Qi L., Gao R., Mei Z., Cheng L., Min W., Kang D., Yu M., Sun Z. (2024). An Investigation on Enhancing the Bonding Properties of PA11-CFRP Interface in Type IV High Pressure Hydrogen Storage Vessel through Nanosecond Pulsed Laser Treatment and Failure Mechanism Research. Int. J. Hydrogen Energy.

[33] Qi L., Min W., Gao R., Li Z., Yu M., Sun Z. (2023). Optimization of Interfacial Bonding Properties between Thermoplastic Liners and Carbon Fiber-reinforced Composites by Atmospheric-pressure Plasma and Failure Mechanism Study. Polym. Compos..

[34] Feki I., Shirinbayan M., Nouira S., Bi R.T., Maeso J.-B., Thomas C., Fitoussi J. (2025). Composites in High-Pressure Hydrogen Storage: A Review of Multiscale Characterization and Mechanical Behavior. Compos. Part C Open Access.

[35] Balasooriya W., Clute C., Schrittesser B., Pinter G. (2022). A Review on Applicability, Limitations, and Improvements of Polymeric Materials in High-Pressure Hydrogen Gas Atmospheres. Polym. Rev..

[36] Kis D.I., Kókai E. (2024). A Review on the Factors of Liner Collapse in Type IV Hydrogen Storage Vessels. Int. J. Hydrogen Energy..

[37] Tamura K., Uno H., Yamada H. (2007). Synthesis and Characterization of Exfoliated Natural Mica/Polymer Nanocomposites. Mater. Sci. Forum.

[38] McNally T., Raymond Murphy W., Lew C.Y., Turner R.J., Brennan G.P. (2003). Polyamide-12 Layered Silicate Nanocomposites by Melt Blending. Polymer.

[39] Fuse N., Kozako M., Tanaka T., Ohki Y. Effects of Mica Fillers on Dielectric Properties of Polyamide Nanocomposites. Proceedings of the CEIDP ’05. 2005 Annual Report Conference on Electrical Insulation and Dielectric Phenomena, 2005.

[40] Pramoda K.P., Liu T., Liu Z., He C., Sue H.-J. (2003). Thermal Degradation Behavior of Polyamide 6/Clay Nanocomposites. Polym. Degrad. Stab..

[41] Dintcheva N., Filippone G., Arrigo R., La mantia F. (2017). paolo Low-Density Polyethylene/Polyamide/Clay Blend Nanocomposites: Effect of Morphology of Clay on Their Photooxidation Resistance. J. Nanomater..

[42] Varga E., Palásti F., Bata A., Kovács P.I. (2024). Comparative Study of Plasma, Laser, and Flame Induced Activation of HDPE Liner Surfaces of Type 4 Hydrogen Vessels. J. Adhes..

[43] (2025). Plastics—Determination of Tensile Properties—Part 2: Test Conditions for Moulding and Extrusion Plastics.

[44] (2023). Plastics—Determination of Charpy Impact Properties—Part 1: Non-Instrumented Impact Test.

[45] Oliver-Ortega H., Méndez J.A., Mutjé P., Tarrés Q., Espinach F.X., Ardanuy M. (2017). Evaluation of Thermal and Thermomechanical Behaviour of Bio-Based Polyamide 11 Based Composites Reinforced with Lignocellulosic Fibres. Polymers.

[46] Su J., Zhang J. (2017). Effect of Treated Mica on Rheological, Cure, Mechanical, and Dielectric Properties of Ethylene Propylene Diene Monomer (EPDM)/Barium Titanate (BaTiO 3)/Mica: ARTICLE. J. Appl. Polym. Sci..

[47] Tang C., Yan H., Li M., Lv Q. (2018). A Novel Phosphorus-Containing Polysiloxane for Fabricating High Performance Electronic Material with Excellent Dielectric and Thermal Properties. J. Mater. Sci. Mater. Electron..

[48] Beran A. (2002). Infrared Spectroscopy of Micas. Rev. Mineral. Geochem..

[49] Kawsihan A., Dissanayake S., Chandrakumara G.T.D., Mantilaka P., Rajapakse R., Pitawala H.M.T.G., de Silva P. (2019). Akaganeite Nanorices Deposited Muscovite Mica Surfaces as Sunlight Active Green Photocatalyst. R. Soc. Open Sci..

[50] Gauvin F., Robert M. (2015). Durability Study of Vinylester/Silicate Nanocomposites for Civil Engineering Applications. Polym. Degrad. Stab..

[51] Lee W.P.C., Wu S., Anariba F., Wu P. (2023). Breaking New Ground in Mica Exfoliation: Harnessing Biaxial Straining Principles through H2 and N2 Intercalation for Enhanced Layer Separation. Mater. Today Adv..

[52] Franceschi G., Brandstetter S., Balajka J., Sokolović I., Pavelec J., Setvin M., Schmid M., Diebold U. (2023). Interaction of Surface Cations of Cleaved Mica with Water in Vapor and Liquid Forms. Faraday Discuss..

[53] Cruz B., Tienne L., Gondim F., Candido L., Marques M., Chaves E. (2020). Influence of the Addition of Multi-walled Carbon Nanotubes on the Thermal and Mechanical Properties of Polyamide-11 before and after Aging Tests. J. Appl. Polym. Sci..

[54] Panaitescu D., Gabor R., Frone A., Vasile E. (2015). Influence of Thermal Treatment on Mechanical and Morphological Characteristics of Polyamide 11/Cellulose Nanofiber Nanocomposites. J. Nanomater..

[55] Yoon K., Polk M., Min B., Schiraldi D. (2004). Structure and Property Study of Nylon-6/Clay Nanocomposite Fiber. Polym. Int..

[56] Varga E., Tóth L., Ádám B., Tajti F., Hansághy P. (2025). Novel Insights into the Morphological Effects of Micron-Scale Inorganic Fillers on Polyethylene Composites. Compos. Commun..

[57] Wu T.-M., Wu J.-Y. (2002). Structural Analysis of Polyamide/Clay Nanocomposites. J. Macromol. Sci. Part B.

[58] Venkataramani S., Lee J.H., Park M.G., Kim S.C. (2008). Structure and Properties of Polyamide-6 & 6/66 Clay Nanocomposites. J. Macromol. Sci. Part A.

[59] Uno H., Tamura K., Yamada H., Umeyama K., Hatta T., Moriyoshi Y. (2009). Preparation and Mechanical Properties of Exfoliated Mica-Polyamide 6 Nanocomposites Using Sericite Mica. Appl. Clay Sci..

[60] Dobrosielska M., Dobrucka R., Brząkalski D., Kozera P., Martyła A., Gabriel E., Kurzydłowski K.J., Przekop R.E. (2023). Polyamide 11 Composites Reinforced with Diatomite Biofiller—Mechanical, Rheological and Crystallization Properties. Polymers.

[61] Zhang Q., Yu M., Fu Q. (2004). Crystal Morphology and Crystallization Kinetics of Polyamide-11/Clay Nanocomposites. Polym. Int..

[62] Verkinderen O., Baeten D., Van Puyvelde P., Goderis B. (2021). The Crystallization of PA11, PA12, and Their Random Copolymers at Increasing Supercooling: From Eutectic Segregation to Mesomorphic Solid Solutions. Polym. Cryst..

[63] Jariyavidyanont K., Focke W., Androsch R. (2016). Crystallization Kinetics of Polyamide 11 in the Presence of Sepiolite and Montmorillonite Nanofillers. Colloid Polym. Sci..

[64] Kolesov I., Androsch R., Mileva D., Lebek W., Benhamida A., Kaci M., Focke W. (2013). Crystallization of a Polyamide 11/Organo-Modified Montmorillonite Nanocomposite at Rapid Cooling. Colloid Polym. Sci..

[65] Sayah N., Smith D.E. (2022). Effect of Process Parameters on Void Distribution, Volume Fraction, and Sphericity within the Bead Microstructure of Large-Area Additive Manufacturing Polymer Composites. Polymers.

[66] Kumari P., Liu T. (2004). Effect of Moisture on the Dynamic Mechanical Relaxation of Polyamide-6/Clay Nanocomposites. J. Polym. Sci. Part B Polym. Phys..

[67] Majka T., Majka M. (2012). The Influence of Maintenance Liquids\’ Absorbence Used in the Automotive Industry on the Mechanical Properties of Polyamide-6/Montmorillonite Nanocomposites. Proceedings of the 16th International Electronic Conference on Synthetic Organic Chemistry session Polymer and Supramolecular Chemistry.

[68] Vlasveld D.P.N., Groenewold J., Bersee H.E.N., Picken S.J. (2005). Moisture Absorption in Polyamide-6 Silicate Nanocomposites and Its Influence on the Mechanical Properties. Polymer.

[69] Taha Z.T., Bata A., Molnár B., Ronkay F. (2025). Impact of Montmorillonite Reinforcement on the Physical Recyclability of Biobased and Petroleum-Based Polyesters. Heliyon.

[70] Dasari A., Yu Z.-Z., Mai Y.-W. (2007). Transcrystalline Regions in the Vicinity of Nanofillers in Polyamide-6. Macromolecules.

[71] Anoukou K., Zaïri F., Naït-Abdelaziz M., Zaoui A., Qu Z., Gloaguen J.M., Lefebvre J.M. (2014). A Micromechanical Model Taking into Account the Contribution of α- and γ-Crystalline Phases in the Stiffening of Polyamide 6-Clay Nanocomposites: A Closed-Formulation Including the Crystal Symmetry. Compos. Part B Eng..

[72] Chen L., Xiang Y., Ke W., Zhang Q., Du R., Fu Q. (2011). Effects of Matrix Molecular Weight on Structure and Reinforcement of High Density Polyethylene/Mica Composites. Chin. J. Polym. Sci..

[73] Fornes T.D., Paul D.R. (2004). Structure and Properties of Nanocomposites Based on Nylon-11 and -12 Compared with Those Based on Nylon-6. Macromolecules.

[74] Ünal H., Mimaroglu A. (2012). Mechanical and Morphological Properties of Mica and Short Glass Fiber Reinforced Polyamide 6 Composites. Int. J. Polym. Mater..

[75] Zhang Y., Yang J.H., Ellis T.S., Shi J. (2006). Crystal Structures and Their Effects on the Properties of Polyamide 12/Clay and Polyamide 6–Polyamide 66/Clay Nanocomposites. J. Appl. Polym. Sci..

[76] Masenelli-Varlot K., Reynaud E., Vigier G., Varlet J. (2002). Mechanical Properties of Clay-reinforced Polyamide. J. Polym. Sci. Part B Polym. Phys..

[77] Mahanwar P.A., Bose S., Tirumalai A.V. (2006). The Influence of Interfacial Adhesion on the Predicted Young’s Modulus of Mica-Reinforced Nylon-6. Polym.-Plast. Technol. Eng..

[78] Ono H. (2024). Micromechanical Analysis for Effective Elastic Moduli and Thermal Expansion Coefficient of Composite Materials Containing Ellipsoidal Fillers Oriented Randomly. Compos. Part C Open Access.

[79] Wang N., Pan X., Wang P., Wang Y., He H., Zeng Y.-J., Zhang L., Li Y., Wang F., Lu B. (2022). Is All Epitaxy on Mica van Der Waals Epitaxy?. Mater. Today Nano.

[80] Liu T., Ping Lim K., Chauhari Tjiu W., Pramoda K.P., Chen Z.-K. (2003). Preparation and Characterization of Nylon 11/Organoclay Nanocomposites. Polymer.

[81] Risite H., El Mabrouk K., Bousmina M., Fassi-Fehri O. (2016). Role of Polyamide 11 Interaction with Clay and Modifier on Thermal, Rheological and Mechanical Properties in Polymer Clay Nanocomposites. J. Nanosci. Nanotechnol..

[82] Bureau M., Denault J., Cole K., Enright G. (2002). The Role of Crystallinity and Reinforcement in the Mechanical Behavior of Polyamide-6/Clay Nanocomposites. Polym. Eng. Sci..

[83] Kis D.I., Bata A., Takács J., Kókai E. (2024). Mechanical Properties of Clay-Reinforced Polyamide 6 Nanocomposite Liner Materials of Type IV Hydrogen Storage Vessels. Nanomaterials.

[84] Bata A., Gerse P., Slezák E., Ronkay F. (2023). Time- and Temperature-Dependent Mechanical and Rheological Behaviours of Injection Moulded Biodegradable Organoclay Nanocomposites. Adv. Ind. Eng. Polym. Res..

[85] Masenelli-Varlot K., Vigier G., Vermogen A., Gauthier C., Cavaillé J.-Y. (2007). Quantitative Structural Characterization of Polymer–Clay Nanocomposites and Discussion of an “Ideal” Microstructure, Leading to the Highest Mechanical Reinforcement. J. Polym. Sci. Part B Polym. Phys..

[86] Vlasveld D.P.N., Jong M., Bersee H., Gotsis A.D., Picken S.J. (2005). The Relation between Rheological and Mechanical Properties of PA6 Nano- and Micro-Composites. Polymer.

[87] da Cruz B.d.S.M., Tienne L.G.P., Gondim F.F., da Silva Candido L., Chaves E.G., Marques M.d.F.V., da Luz F.S., Monteiro S.N. (2022). Graphene Nanoplatelets Reinforced Polyamide-11 Nanocomposites Thermal Stability and Aging for Application in Flexible Pipelines. J. Mater. Res. Technol..

